# Insight into durum wheat *Lpx-B1*: a small gene family coding for the lipoxygenase responsible for carotenoid bleaching in mature grains

**DOI:** 10.1186/1471-2229-10-263

**Published:** 2010-11-26

**Authors:** Angelo Verlotta, Vanessa De Simone, Anna M Mastrangelo, Luigi Cattivelli, Roberto Papa, Daniela Trono

**Affiliations:** 1 CRA-Cereal Research Centre, S.S. 16, Km 675 - 71122 Foggia, Italy

## Abstract

**Background:**

The yellow colour of pasta products is one of the main criteria used by consumers to assess pasta quality. This character is due to the presence of carotenoid pigments in semolina. During pasta processing, oxidative degradation of carotenoid pigments occurs mainly due to lipoxygenase (LOX). In durum wheat (*Triticum durum *Desf.), two *Lpx-1 *genes have been identified on chromosome 4B, *Lpx-B1.1 *and *Lpx-B1.2*, and evidences have been reported that the deletion of *Lpx-B1.1 *is associated with a strong reduction in LOX activity in semolina. In the present study, we characterised the *Lpx-B1 *gene family identified in a durum wheat germplasm collection and related the distribution and expression of the *Lpx-B1 *genes and alleles to variations in LOX activity in the mature grains.

**Results:**

In addition to the already known *Lpx-B1.1 *and *Lpx-B1.2 *genes, a new gene was identified, *Lpx-B1.3*, along with three different *Lpx-B1.1 *alleles, *Lpx-B1.1a*, *Lpx-B1.1b *and the partially deleted *Lpx-B1.1c*. Screening of the germplasm collection showed that all of the genotypes have one of the three *Lpx-B1.1 *alleles, associated with either *Lpx-B1.2 *or *Lpx-B1.3*, thus showing that in this collection the two genes are alternatives. Therefore, based on *Lpx-B1 *distribution, three different haplotypes were distinguished: haplotype I, carrying *Lpx-B1.3 *and the *Lpx-B1.1b *allele; haplotype II carrying *Lpx-B1.2 *and the *Lpx-B1.1a *allele; and haplotype III carrying *Lpx-B1.2 *and the *Lpx-B1.1c *allele. Determination of *Lpx-B1 *transcript abundance and total LOX activity in mature grains revealed differences among these three haplotypes: haplotypes I, II and III showed high, intermediate and low levels, respectively, of functional *Lpx-B1 *transcripts and enzymatic activity.

**Conclusions:**

In this germplasm collection, the *Lpx-B1 *gene family accounts for most of the total LOX activity in the mature grains. Information on these *Lpx-B1 *haplotypes provides significant improvement for prediction of LOX-1 activity levels in mature grains, and will therefore help in breeding programmes aimed at selection of new durum wheat genotypes with higher carotenoid contents in their end products.

## Background

The yellow colour is an important parameter in the definition of pasta quality, and it is therefore a target for durum wheat (*Triticum durum *Desf.) breeding. This character arises from carotenoid pigments in semolina, which are mainly free and esterified lutein. As well as its role as an important aesthetic parameter of pasta products, lutein has high nutritional value. Known mostly for its importance in reducing the risk for ocular diseases, including age-related macular degeneration and cataracts, lutein consumption has been recently related to a reduced risk for developing cardiovascular disease and several types of cancer [1]. On this basis, enhancement of the lutein content in a widely consumed food such as pasta will be beneficial towards the maintenance of human health, particularly with the current low daily intake of lutein compared to the daily recommended dose.

However, during pasta processing there is often a loss of colour as a consequence of pigment oxidation. Although other enzymes present in durum wheat semolina can contribute to carotenoid bleaching, such as peroxidases and polyphenol oxidases, the major role appears to be that of the lipoxygenase-linoleate system [2-4].

Lipoxygenase (LOX, linoleate:oxygen oxidoreductase; EC 1.13.11.12) is a class of non-heme iron-containing dioxygenases that catalyses the positional and specific dioxygenation of polyunsaturated fatty acids that contain 1,4-*cis,cis *pentadiene structures to produce the corresponding hydroperoxides. In plants, products of the LOX reaction have been shown to have roles in several processes, such as vegetative growth, wounding, response to herbivore and pathogen attack and also mobilisation of storage lipids during germination [5]. In durum wheat semolina, radicals produced during the intermediate states of linoleate hydroperoxidation can cause oxidation of carotenoid pigments, and consequently a loss of the yellow colour in pasta products [5].

The occurrence of LOX enzymes in cereal grains has been well documented for barley (*Hordeum vulgare *L.). Two different isoforms, LOX-1 and LOX-2, have been purified and characterised [6]: LOX-1 contributes almost exclusively to the total LOX activity in quiescent grains, whereas LOX-2 activity increases rapidly during germination [7]. Three cDNA sequences encoding the LOX proteins have been isolated from barley grains, and the chromosomal locations of their corresponding genes determined: the *LoxA *and *LoxC *clones correspond to the LOX-1 and LOX-2 isoforms, respectively [8], and a third clone, known as *LoxB *[9], encodes a LOX isoform that has not been identified to date. The *LoxA *and *LoxB *loci are tightly linked (1 cM) and map to chromosome 4HS, whereas *LoxC *maps to the long arm of chromosome 5H [9]. The *LoxB *transcript has been mainly detected in germinating grains, although at levels significantly lower than for *LoxA *and *LoxC*, with no *LoxB *transcript in mature grains [9].

In durum wheat the LOX genes are located in regions collinear with barley, thus suggesting that they are orthologous: two *Lpx-2 *sequences that correspond to the barley *LoxC *gene have been identified and assigned to the group 5 chromosomes, whereas two sequences designated *Lpx-3 *and showing higher similarity with barley *LoxB *have been mapped on the group 4 chromosomes [10]. As far as durum wheat genes orthologous to barley *LoxA*, Hessler et al. highlighted three natural *Lpx-1 *gene variants in durum wheat that have arisen from the instability of a miniature inverted-repeat transposable element (MITE) in the last intron [11]. The MITE has been shown to be complete (C7.2.1), partial (J4.2), or even absent (J2.2/C5.36.2), and this polymorphism has allowed mapping of the *Lpx-1 *sequences to chromosome 4BS. Based on similarities among the three sequences and on their distribution among cultivars, the authors hypothesized that the C7.2.1 and the J4.2 sequences represent alleles at the same *Lpx-1 *locus on the short arm of chromosome 4B, whereas the J2.2 and C5.36.2 sequences correspond to a gene at a different *Lpx-1 *locus. This hypothesis has been successively supported by findings of Carrera et al. [10], who highlighted two sequences, DQ474240 and DQ474241, that corresponded to the sequences with and without the MITE, respectively, previously identified by Hessler et al. [11]. These sequences were shown to map at two linked *Lpx-B1 *loci on wheat chromosome 4B, which were designated *Lpx-B1.1 *and *Lpx-B1.2*, respectively. The same study also reported an allelic variation for the deletion of the *Lpx-B1.1 *copy; authors' findings demonstrated that the deletion is associated with a strong reduction in LOX activity in semolina extracts. A partially deleted copy of *Lpx-1 *has also been found on chromosome 4A [12].

The variability in carotenoid content and LOX activity of durum wheat germplasm was recently assessed in a large collection, and the contribution of the *Lpx *genes in the determination of pigment loss during pasta processing was also determined [4]. This study confirmed the correlation between high/low LOX activity levels in semolina and the presence/absence of the *Lpx-B1.1 *locus, and demonstrated that the *Lpx *genes are differently expressed during seed maturation, with *Lpx-1 *transcripts being the most abundant in mature grains.

Here we analysed the role of *Lpx-1 *in the determination of LOX activity in mature durum wheat grains. An in-depth characterization of five genes and alleles at the *Lpx-B1 *locus present in a germplasm collection was performed. The full-length sequences of the *Lpx-B1 *genes/alleles were isolated and characterized. On the basis of sequence polymorphisms and map positions, a new gene designated *Lpx-B1.3 *was identified in addition to the already known *Lpx-B1.1 *and *Lpx-B1.2 *genes, and three different alleles at the *Lpx-B1.1 *locus were distinguished. According to the distribution of the *Lpx-B1 *genes/alleles in the germplasm collection, three distinct groups were identified that correspond to three different haplotypes and are characterized by different *Lpx-B1 *expression profiles and LOX activity in mature grains.

## Results

### Identification and characterisation of genes and alleles at the *Lpx-B1 *locus of durum wheat

To gain further insight into the organisation of the *Lpx-B1 *locus, we investigated whether this locus includes genes and/or alleles other than those already identified, and evaluated the consequent distribution in a large durum wheat germplasm collection.

**Figure 1:**
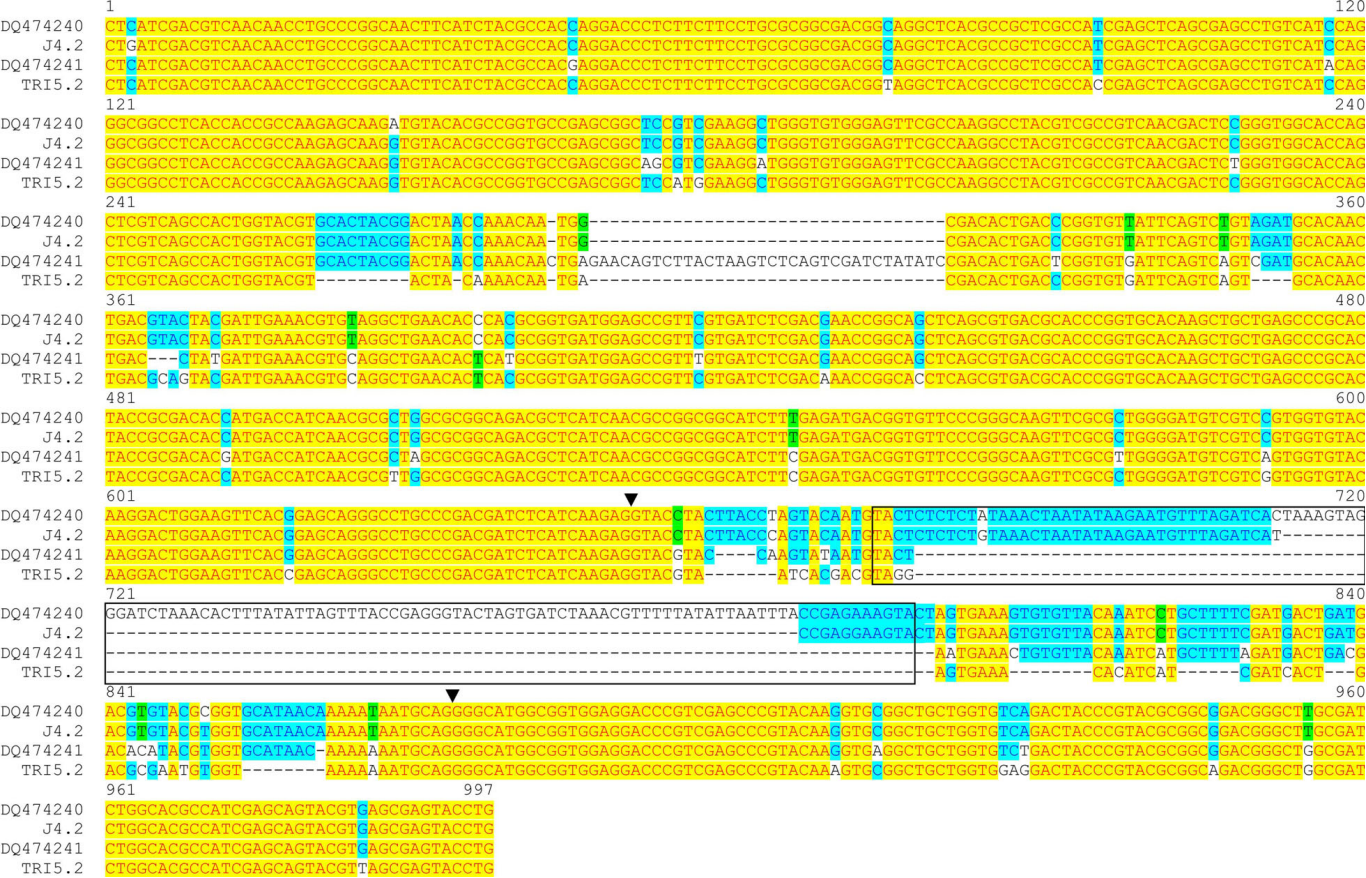
**Alignment of the four partial genomic sequences isolated from the collection of durum wheat genotypes**. Arrows indicate the start and stop of the intron region that contains the MITE (boxed sequence) discovered by Hessler et al. [11].

We surveyed 85 durum wheat genotypes by performing an amplification reaction on genomic DNA with the primer pair LOXA-L1/R1 (Table 1) previously employed by Carrera et al. to preferentially amplify sequences at the *Lpx-B1 *locus [10]. The fragments obtained were cloned and sequenced to prove their identity. Excluding singletons, four different sequences were distinguished (Figure 1). Two sequences corresponded to DQ474240 (*Lpx-B1.1*) and DQ474241 (*Lpx-B1.2*); the presence of the MITE in the last intron of the *Lpx-B1.1 *gene and its absence in the *Lpx-B1.2 *gene allowed size discrimination of these two amplified fragments, with lengths of 962 bp and 867 bp, respectively. A third sequence corresponded to J4.2 (888 bp long), as previously identified by Hessler et al. [11]; in line with previous findings, this sequence shared 99.9% identity with DQ474240 in the coding region, and carried a partial sequence of the MITE in the last intron, which corresponded to the first half of the element and a final 11-bp inverted repeat. As well as these already known sequences, a fourth new sequence was identified: TRI5.2. In the coding region, TRI5.2 shared 97.0% and 95.6% identity with the fragments at the *Lpx-B1.1 *(DQ474240 and J4.2) and *Lpx-B1.2 *(DQ474241) loci, respectively. Several deletions in the intron regions of this sequence, one of which caused complete removal of the MITE, allowed size discrimination between this fragment (794 bp long) and the other three fragments.

**Table 1:** Primer pairs used to amplify Lpx-B1 genes/alleles and transcripts, and PCR product sizes

	Forward primer (5'→3')	Reverse primer (5'→3')	Product size (bp)	
***Full length***			***Gene***	***Transcript***
*All Lpx-B1s*	CCAAGATGATACTGGGCGGGC	CGCCGCCTTGCCGTGGTTGG	1558-4320	1154-2369
				
***Fragment***			***Gene***	***Transcript***
*Lpx B1.1a/Lpx B1.1b/* *Lpx-B1.2/Lpx-B1.3 *	L1, CTGATCGACGTCAACAAC^a^	R1, CAGGTACTCGCTCACGTA^a^	962/888/ 867/794^b^	--
				
	**GCAGGCGCTGGAAAGCAACAGGC**^c^	**ACTCCGCGTACTCGTCCGTCCCG**^c^	**1312/1238**^b^	**992**
*Lpx B1.1a/Lpx-B1.1b*	ACGCCGGTGCCGAGCGGCTC	GCGCTCTAACTCCGCGTACTCG	1106/1032^b^	786
	CCACGCGGTGATGGAGCCGTTC	GCGTGTCGCGCTGACCCAGGTAC	1023/949^b^	800
				
*Lpx-B1.1c*	**CCAAGATGATACTGGGCGGGC**^c^	**CGCCGCCTTGCCGTGGTTGG**^c^	1558	1154
				
	**TACACGCCGGTGCCGAGCGGCAG**^c^	**CGTGTCACGCTGCCCGAGGTAGAG**^c^	**1137**	**912**
*Lpx B1.2*	CCAGGCGGACCAGCAGGCGTGGC	ATGTACAACCGGTTGCTTTCAAGG	732	225
	TCAACGCCGGCGGCATCTTC	CGGACCGTTGCGGTTCAACAGCTG	890	794
				
	**CCGGTGCCGAGCGGCTCCATG**^c^	**CGGTTCGGGAGGAACCCCGCGTAG**^c^	**871**	**729**
*Lpx-B1.3*	GCCGGCGGTGCGCACATACGTG	GTCTGCCGCGTACGGGTAGTCCTC	1585	1056
	GGTAGGCTCACGCCGCTCGCCAC	GCACCGGGTGCGTCACGCTGAGG	339	256
				
*17S rRNA*	**GCTCGTAGTTGGACCTTGG**^c^	**GTATCTGATCGTCTTCGAGC**^c^	**--**	**382**

^a^This is the LOXA-L1/R1 primer pair previously used by Carrera et al. [10]. ^b^The different amplicon sizes are due to the length polymorphism of the MITE in the intron region. ^c^Primer pairs highlighted in bold were those used for Figures 3B and 5.

We then isolated and characterised the full-length genomic sequences corresponding to these four *Lpx-B1 *fragments. This approach allowed isolation of four sequences [GenBank: HM126466, HM126467, HM126468, HM126469] that ranged between 4,062 bp and 4,263 bp (from start to stop codon) (Table 2), and included a portion that was 100% identical to the DQ474240, DQ474241, J4.2 and TRI5.2 sequences. In addition to the full-length sequences that corresponded to the four fragments reported in Figure 1, a shorter sequence was also isolated, of 1,505 bp (from start to stop codon) [GenBank: HM126470] (Table 2).

**Table 2:** General features of the three haplotypes at gene, transcript and activity levels

Haplotype	Nomenclature	Gene/allele		Transcript		**LOX activity**^**a **^**(E.U./g of dry weight)**	
		
		**GenBank accession n**.	Length (bp)	**GenBank accession n**.	Length (bp)	Linoleate hydroperoxidation	**β-carotene bleaching × 10**^**-2**^
I	*Lpx-B1.1b* *Lpx-B1.3*	HM126468 HM126469	4,172 4,062	HM126473 HM126469	2,586 2,586	4.60 ± 2.38^A^	5.76 ± 1.50^A^
							
II	*Lpx-B1.1a* *Lpx-B1.2*	HM126466 HM126467	4,246 4,263	HM126471 HM126472	2,586 2,586	2.59 ± 1.08^B^	3.34 ± 0.65^B^
							
III	*Lpx-B1.1c* *Lpx-B1.2*^*b*^	HM126470 HM126467	1,505 4,263	HM126475 HM126472	1,101 2,586	0.12±0.09^c^	0.47±0.23^c^

^a^Values are expressed as means of the three field replications ± S.D.; for each activity the different uppercase letters correspond to significantly different values (*P *< 0.05). ^b^The full-length of the *Lpx-B1.2 *gene isolated from the haplotype III cultivar Ofanto was found to be 100% identical to that isolated from the haplotype II cultivar Primadur.

To determine the genomic structure of the five *Lpx-B1 *sequences, comparisons were made with the corresponding expressed sequences (see below) and with the rice (*Oryza sativa *L.) *9-LOX1 *gene [GenBank: AB099850]. This latter was chosen because it is phylogenetically closely related to barley *LoxA *(L35931) [13,14] and is located on rice chromosome 3, which shows synteny with a large portion of wheat chromosome 4B. In particular, a syntenic correspondence with rice chromosome 3 was seen for the 4BS8-0.57-0.81 bin, in which the ESTs corresponding to the *Lpx-B1 *sequences were physically mapped (GrainGenes website http://wheat.pw.usda.gov/wEST/binmaps/wheat4_rice.html).

**Figure 2:**
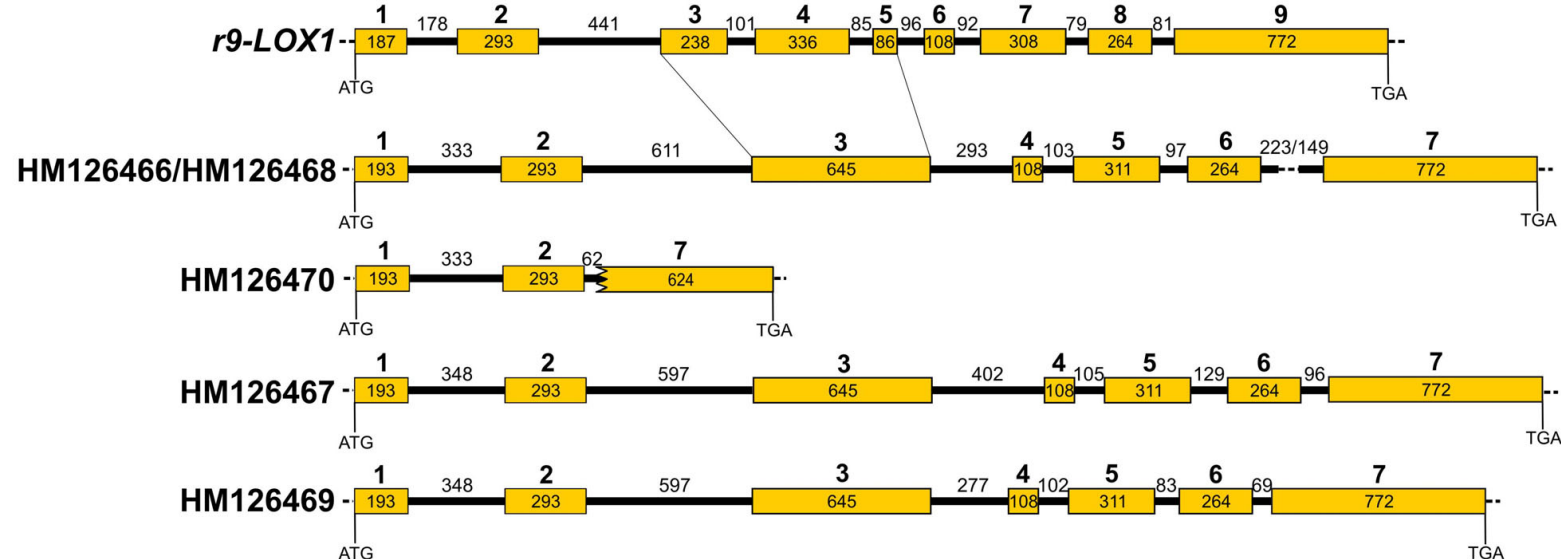
**Schematic representation of the structures of the *Lpx-B1 *genes**. The structures of the *Lpx-B1 *genes were deduced by comparisons of the genomic sequences with the rice *r9-LOX1 *gene (AB099850) and with the corresponding transcripts (see below). Filled boxes represent exons and lines represent introns. The dotted line in the last intron of the HM126466 and HM126468 sequences represents the MITE length polymorphism. The translation start and the stop codons are also indicated.

As shown in Figure 2, the four longer sequences of the complete *Lpx-B1 *genes/alleles shared a common structure with rice *9-LOX1*, except that rice exons 3 to 5 were joined in the durum wheat genes; consequently, the durum wheat genes comprised seven exons and six introns. In contrast, the shorter sequence was a partially deleted gene, which included only the region from the first exon to the start of the second intron, with a large part of the last exon. As the LOXA-L1 and LOXA-R1 primers fall into exons 5 and 7, respectively, no fragment corresponding to the partially deleted gene was identified in the experiment reported in Figure 1.

While the length of the exons was highly conserved among the *Lpx-B1 *genes/alleles, the introns showed some degree of variability. In particular, the HM126466 and HM126468 sequences were characterised by the same intron length, with the exception of intron 6, the length of which differed between the two sequences due to the polymorphism of the MITE length. As well as the common exon/intron structure, the two sequences shared high identity (99.4%). This is in line with the hypothesis of Hessler et al. [11], that the two sequences represent alleles at the same *Lpx-B1.1 *locus. In the light of this, we have designated the HM126466 sequence as *Lpx-B1.1a *and the HM126468 sequence as *Lpx-B1.1b*. The partially deleted gene corresponding to the HM126470 sequence was closely related to the *Lpx-B1.1 *alleles: in the overlapping regions it showed the same exon/intron structure, with 99% identity.

The HM126467 sequence, that had previously been assigned to the *Lpx-B1.2 *locus, and the HM126469 sequence, that was identified in the present study, showed different intron lengths, both with each other (with the exception of introns 1 and 2) and with the *Lpx-B1.1 *alleles. On the basis of percentage identities, these two sequences were more similar to each other than to the alleles at the *Lpx-B1.1 *locus (92.6% *vs *83.0% to 85.7%).

### Genetic mapping of the *Lpx-B1 *genes and alleles

**Figure 3:**
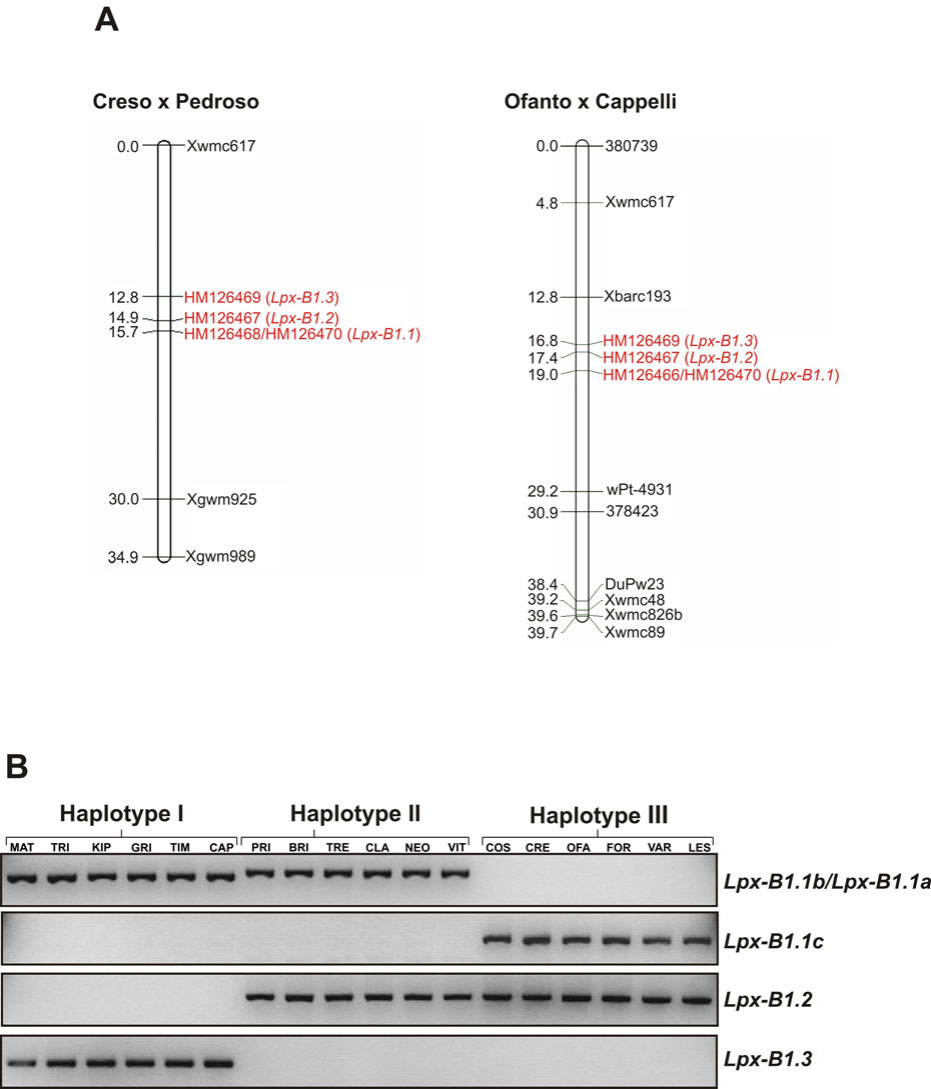
**Chromosomal mapping and analysis of the distribution among the genotypes of the *Lpx-B1 *genes and alleles**. (A) The genetic linkage groups of chromosome 4BS from Creso × Pedroso and Ofanto × Cappelli RIL populations. The genetic distances of markers are indicated in cM to the left of the chromosome bars. (B) Distribution of the *Lpx-B1 *genes and alleles in 18 durum wheat cultivars of the germplasm collection. Representative gel separation of genomic DNA extracted from leaves and amplified using gene and allele specific primer pairs (highlighted in bold in Table 1), according to conditions described in Methods. MAT, Matarese; TRI, Trinakria; KIP, Kiperounda; GRI, Grifoni; TIM, Timilia; CAP, Capeiti 8; PRI, Primadur; BRI, Brindur; TRE, Tresor; CLA, Claudio; NEO, Neodur; VIT, Vitromax; COS, Cosmodur; CRE, Creso; OFA, Ofanto; FOR, Fortore; VAR, Varano; LES, Lesina. The gel separation is in inverted colours.

To determine the chromosomal positions of the genes/alleles identified in the present study, linkage analysis was carried out on two recombinant inbred line (RIL) populations derived from the crosses between Creso × Pedroso and Ofanto × Cappelli. These populations were chosen because in both cases the parental genotypes were characterised by different *Lpx-B1 *genes/alleles: Creso and Ofanto carried the sequence HM126467 (*Lpx-B1.2*) and the new sequence HM126470, Pedroso and Cappelli carried, respectively, the sequence HM126468 (*Lpx-B1.1b*) and HM126466 (*Lpx-B1.1a*), and both of them carried the new sequence HM126469. As shown in Figure 3A, in both of the RIL populations, all of the four genes/alleles mapped to the short arm of chromosome 4B, about 13 cM proximal to the microsatellite marker *Xwmc617*, where the map position of the *Lpx-B1 *locus was previously determined [10,15]. Markers in the telomeric region of chromosome 4BS showed significant (*P *< 0.01) segregation distortion in the Ofanto × Cappelli map. In line with previous findings, HM126466/HM126468 and HM126467 mapped as two separate loci, *Lpx-B1.1 *and *Lpx-B1.2*, respectively. The new sequence HM126470 was seen to co-segregate with HM126466/HM126468, thus suggesting that it represents a third allelic variant at the *Lpx-B1.1 *locus, referred to hereafter as *Lpx-B1.1c*; this might correspond to the deletion previously identified by Carrera et al. [10]. The HM126469 sequence was located at a different locus, and this new genomic position was designated *Lpx-B1.3*.

The mapping results were consistent with those obtained from the screening carried out to determine the distribution of the *Lpx-B1 *genes and alleles in the germplasm collection. As shown in Table 3, no recombinant genotypes were found among *Lpx-B1.1a*, *Lpx-B1.1b *and *Lpx-B1.1c*, as expected from alleles at the same locus. All of the genotypes had one of the three alleles at the *Lpx-B1.1 *locus, although none of them showed both *Lpx-B1.2 *and *Lpx-B1.3*. These data suggest that other alleles that were not detected with the primers used might exist at the *Lpx-B1.2 *and *Lpx-B1.3 *loci in the durum wheat germplasm. The haplotype analysis at the *Lpx-B1 *locus highlighted three different groups of genotypes, an example of which is shown in Figure 3B. Haplotype I included exclusively old genotypes released before 1971, carrying the *Lpx-B1.1b *allele associated with the *Lpx-B1.3 *gene, while haplotypes II and III comprised genotypes carrying *Lpx-B1.1a *and the *Lpx-B1.1c*, respectively, associated with the *Lpx-B1.2 *gene (Table 2). The only exception to this classification was the old genotype Cappelli, that presented the *Lpx-B1.1a *allele associated with the *Lpx-B1.3 *gene.

**Table 3:** Distribution of the Lpx-B1 genes and alleles among the 85 durum wheat genotypes.

Genotypes	Country of origin	Locus				
		*Lpx-B1.1*			*Lpx-B1.2*	*Lpx-B1.3*
				
**Old (pre-1971)**		*Lpx-B1.1a*	*Lpx-B1.1b*	*Lpx-B1.1c*		

Trinakria	Italy		✔			✔

Timilia	Italy		✔			✔

Grifoni	Italy		✔			✔

Capeiti 8	Italy		✔			✔

Aziziah	Italy		✔			✔

Kiperounda	Libya		✔			✔

Polesine	Cyprus		✔			✔

Taganrog	Italy	✔			✔	

Russello	USSR	✔			✔	

Cappelli	Italy	✔			✔	

Cannizzara	Italy	✔				✔

Trinakria	Italy			✔	✔	

**Intermediate (1971-1990)**						

Primadur	France	✔			✔	

Tresor	France	✔			✔	

Neodur	France	✔			✔	

Maghrebi 72	Tunisia	✔			✔	

Karel	Italy	✔			✔	

Latino	Italy	✔			✔	

Duilio	Italy	✔			✔	

Valnova	Italy			✔	✔	

Produra	USA			✔	✔	

Valgerardo	Italy			✔	✔	

Creso	Italy			✔	✔	

Grazia	Italy			✔	✔	

Ofanto	Italy			✔	✔	

Valforte	Italy			✔	✔	

Simeto	Italy			✔	✔	

**Modern (1991-2005)**						

Marco	Italy	✔			✔	

Gianni	Italy	✔			✔	

Bronte	Italy	✔			✔	

Rusticano	Italy	✔			✔	

Tiziana	Italy	✔			✔	

Parsifal	France	✔			✔	

Arcobaleno	Italy/Spain	✔			✔	

Svevo	Italy	✔			✔	

Giotto	Italy	✔			✔	

Iride	Italy	✔			✔	

Dupri	Italy	✔			✔	

Italo	Italy	✔			✔	

Verde	Unknown	✔			✔	

Radioso	Italy	✔			✔	

Brindur	France	✔			✔	

Claudio	Italy	✔			✔	

Torrebianca	Italy	✔			✔	

Vitromax	Italy/Spain	✔			✔	

Nefer	France	✔			✔	

L29	Italy	✔			✔	

L83	Italy	✔			✔	

5Bil-90	Italy	✔			✔	

5Bil-85	Italy	✔			✔	

5Bil-28	Italy	✔			✔	

5Bil-46	Italy	✔			✔	

CTA440	Italy	✔			✔	

Messapia	Italy			✔	✔	

Gargano	Italy			✔	✔	

Carpio	Italy			✔	✔	

Saadi	France			✔	✔	

Cirillo	Italy			✔	✔	

Adamello	Italy			✔	✔	

Colosseo	Italy			✔	✔	

Quadrato	Italy			✔	✔	

Ciccio	Italy			✔	✔	

Vesuvio	Italy			✔	✔	

Platani	Italy			✔	✔	

Colorado	Italy/USA			✔	✔	

Giusto	Italy			✔	✔	

Solex	Italy			✔	✔	

S. Carlo	Italy			✔	✔	

Bradano	Italy			✔	✔	

Preco	Italy			✔	✔	

Cosmodur	France			✔	✔	

Fortore	Italy			✔	✔	

Varano	Italy			✔	✔	

Lesina	Italy			✔	✔	

Zenit	Italy			✔	✔	

L89	Italy			✔	✔	

CTA432	Italy			✔	✔	

CTA503	Italy			✔	✔	

CTA529	Italy			✔	✔	

L91	Italy			✔	✔	

CTA491	Italy			✔	✔	

L95	Italy			✔	✔	

CTA478	Italy			✔	✔	

L38	Italy			✔	✔	

L102	Italy			✔	✔	

### Isolation and characterisation of full-length *Lpx-B1 *cDNAs from mature grains

The full-length *Lpx-B1 *transcripts were isolated after amplification of cDNA from mature grains. Five expressed sequences were isolated that were identical to the coding sequences deduced from the corresponding *Lpx-B1 *genes and alleles, thus demonstrating that they were all actively transcribed, with four of them potentially encoding functional LOX-1 isoforms. Three of the expressed sequences [GenBank: HM126471, HM126473, HM126475] corresponded exactly to the coding sequences of the three alleles at the *Lpx-B1.1 *locus, *Lpx-B1.1a*, *Lpx-B1.1b *and *Lpx-B1.1c *(respectively) (Table 2). For the deleted allele, it is important to note that the lack of the canonical acceptor site in the 5'-portion of the last exon activates a cryptic AG splice site that corresponds to positions + 8 and + 9 of the last exon; this results in a mature mRNA that lacks the first nine nucleotides of the last exon. The other two sequences [GenBank: HM126472, HM126474] were 100% identical to the coding sequences deduced from the *Lpx-B1.2 *and *Lpx-B1.3 *genes (respectively) (Table 2). The expressed sequences that corresponded to the complete genes were 2,586 bp long (from start to stop codon), whereas the deleted variant was 1,101 bp long, as it lacked a central portion of 1,485 bp, from nucleotide 487 to 1,971 (Table 2).

**Figure 4:**
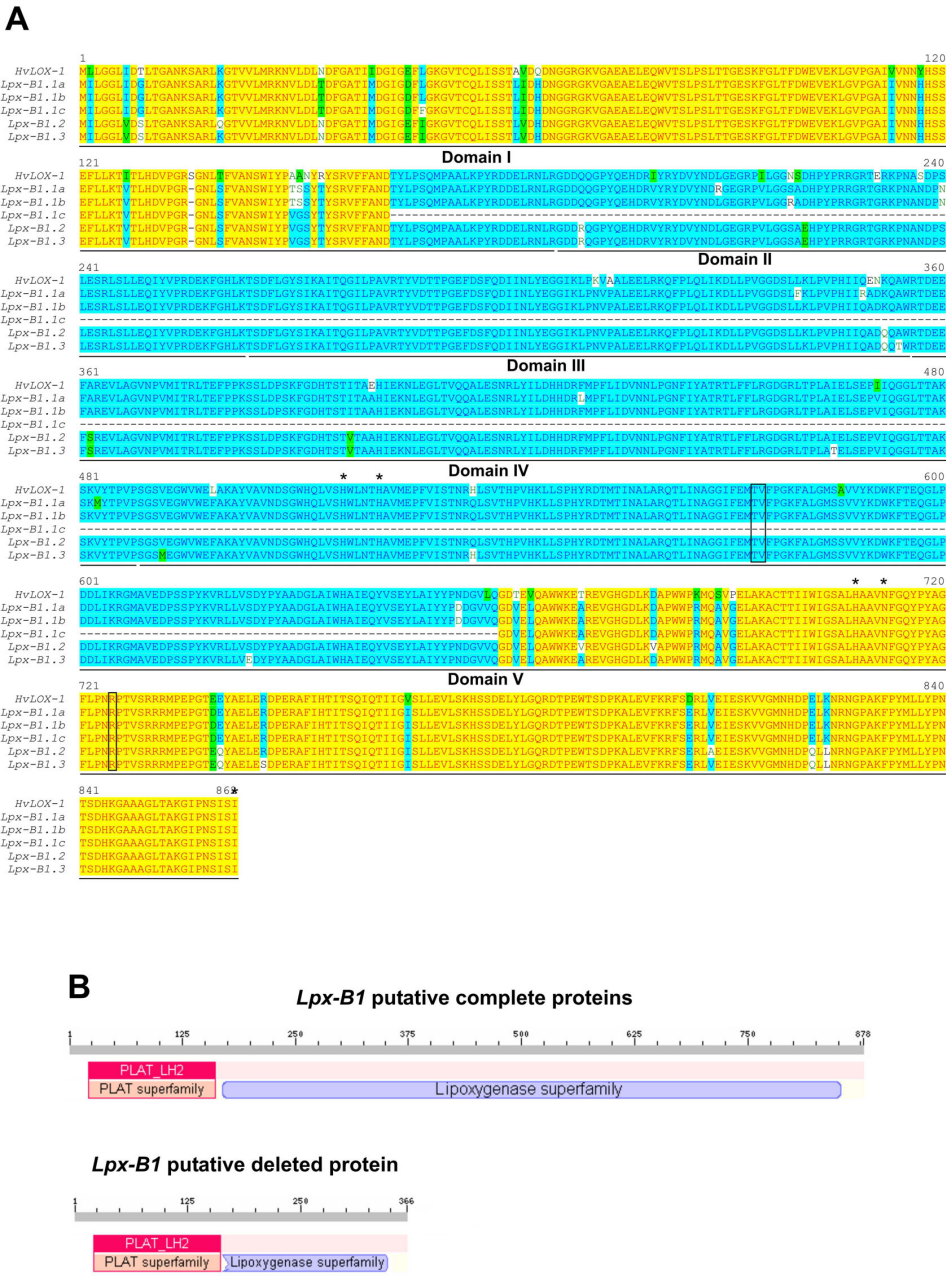
**Analysis of the deduced amino-acid sequences of durum wheat LOX-1s**. (A) Protein sequence alignments between barley LOX-1 (*Hv*LOX-1) [GenBank: L35931] and durum wheat LOX-1s deduced from the expressed sequences. Conserved domains (from I to V) as assigned by Minor et al. [17] are underlined. Asterisks indicate residues required for iron binding. The 9-LOX-specific TV/R motif is boxed. The alignments were performed using Vector NTI AlignX software (Ver. 9.0, Invitrogen). (B) N-terminal PLAT domain and typical LOX domain in both the complete and deleted variants of durum wheat LOX-1s, as identified by the NCBI Conserved Domain Search Database (http://www.ncbi.nlm.nih.gov/Structure/cdd/cdd.shtml).

As shown in the structure analyses of the deduced protein sequences in Figure 4, the complete transcripts harboured an open reading frame that encoded a presumed translation product of 861 amino-acid residues, with a molecular mass of about 96 kDa and a pI around 5.8, estimated using the ProtParam tool [16]. Highly conserved regions were identified through a comparison with the best characterised plant LOX protein: soybean (*Glycine max *L. Merr.) LOX-1 [17]. Three main features were identified: (i) the PLAT/LH2 domain (domain I) in the N-terminal region, which consists of a β-barrel structure that can facilitate access to the substrate; (ii) the LOX domain PFAM00305 (domains II-V), which is typical of the lipoxygenase superfamily and includes the conserved regions that are important for substrate and oxygen binding; and (iii) the residues involved in iron binding (His 516, His 521, His 707, Asn 711 and Ile 861). As expected, the active site had the TV/R motif, which defines the 9-LOX region specificity in plant 9-LOX isoforms [18].

For the deleted transcript, the deletion did not interrupt the frame, thus harbouring an open reading frame that coded for a putative protein product of 366 amino-acid residues (Figures 4A and 4B) with a molecular mass of about 40 kDa and a pI of 5.79 [16]. The presumed translation product maintained the β-barrel structure, but lacked a central region spanning from domain II to the first half of domain V. As this missing part of the protein is essential for catalysis, it is likely that even if produced, this protein will be inactive.

As shown in Table 4, the three *Lpx-B1.1 *alleles shared high identity at both transcript and amino-acid level (99.2-99.3%, in bold). The complete *Lpx-B1.1a *and *Lpx-B1.1b *transcripts shared 11 single nucleotide polymorphisms (SNPs), 5 of which were silent mutations, and 9 and 12 SNPs, respectively, with the deleted *Lpx-B1.1c *transcript, of which only 3 gave rise to amino-acid substitutions.

**Table 4:** Sequence comparisons among the five durum wheat Lpx-B1 transcripts (above the diagonal) and among their deduced amino-acid sequences (below the diagonal) (% irrespective of sequence length). The two groups that include putative proteins sharing the highest identity with each other are highlighted in bold (Lpx-B1.1s) and in bold italic (Lpx-B1.2 and Lpx-B1.3)

Locus		*Lpx-B1.1*			*Lpx-B1.2*	*Lpx-B1.3*
		
	Allele	*Lpx-B1.1a*	*Lpx-B1.1b*	*Lpx-B1.1c*		
	*Lpx-B1.1a*	**100**	99.6	99.2	95.9	95.7
*Lpx-B1.1*	*Lpx-B1.1b*	**99.3**	**100**	99.0	95.9	95.8
	*Lpx-B1.1c*	99.2	**99.2**	**100**	95.7	95.6
*Lpx-B1.2*		96.7	97.2	97.3	***100***	97.6
*Lpx-B1.3*		96.4	96.9	96.7	***99.0***	***100***

For transcripts that corresponded to the *Lpx-B1.2 *and *Lpx-B1.3 *genes, these were more closely related to each other than to the *Lpx-B1.1 *alleles (97.6% *vs *95.6% to 95.9% identity). The degree of identity was even higher at the amino-acid level (99.0%, in bold italic), as only 10 out of the 63 SNPs shared by the two expressed sequences gave rise to amino-acid substitutions.

### Expression analysis of the *Lpx-B1 *genes and alleles in mature grains

**Figure 5:**
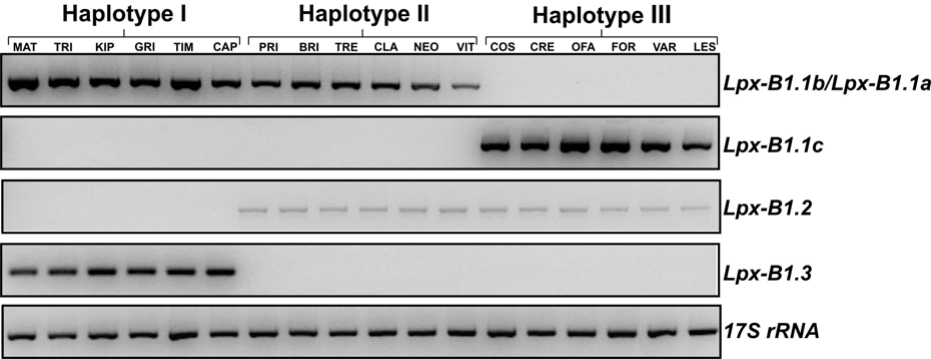
**Expression analysis of *Lpx-B1 *genes and alleles**. Total RNA was isolated from the mature grains of the 18 durum wheat cultivars of the germplasm collection and subjected to semi-quantitative RT-PCR analysis using the same specific primer pairs used for Figure 3B, and amplification conditions described in Methods. Normalisation of the PCR reactions was performed using primers designed on the wheat 17S rRNA constitutive genes. For cultivar abbreviations, see legend to Figure 3B. The gel separation is in inverted colours.

To determine the expression levels of the *Lpx-B1 *genes and alleles, semi-quantitative RT-PCR was carried out on the cDNA isolated from mature grains of the same 18 durum wheat cultivars used in Figure 3B. As shown in Figure 5, the distribution (presence/absence) among the cultivars of the five transcripts corresponded precisely to that at the gene level. The *Lpx-B1.1 *alleles were expressed at high levels. Particularly high levels of expression were seen for the *Lpx-B1.1b *and *Lpx-B1.1c *alleles in haplotypes I and III, respectively, whereas slightly lower levels were seen for the transcript corresponding to the *Lpx-B1.1a *allele in haplotype II. High levels of expression were also seen for the *Lpx-B1.3 *gene in haplotype I, while the *Lpx-B1.2 *gene in haplotypes II and III was expressed at low levels.

### Evaluation of LOX-catalysed linoleate hydroperoxidation and β-carotene bleaching in mature grains

**Figure 6:**
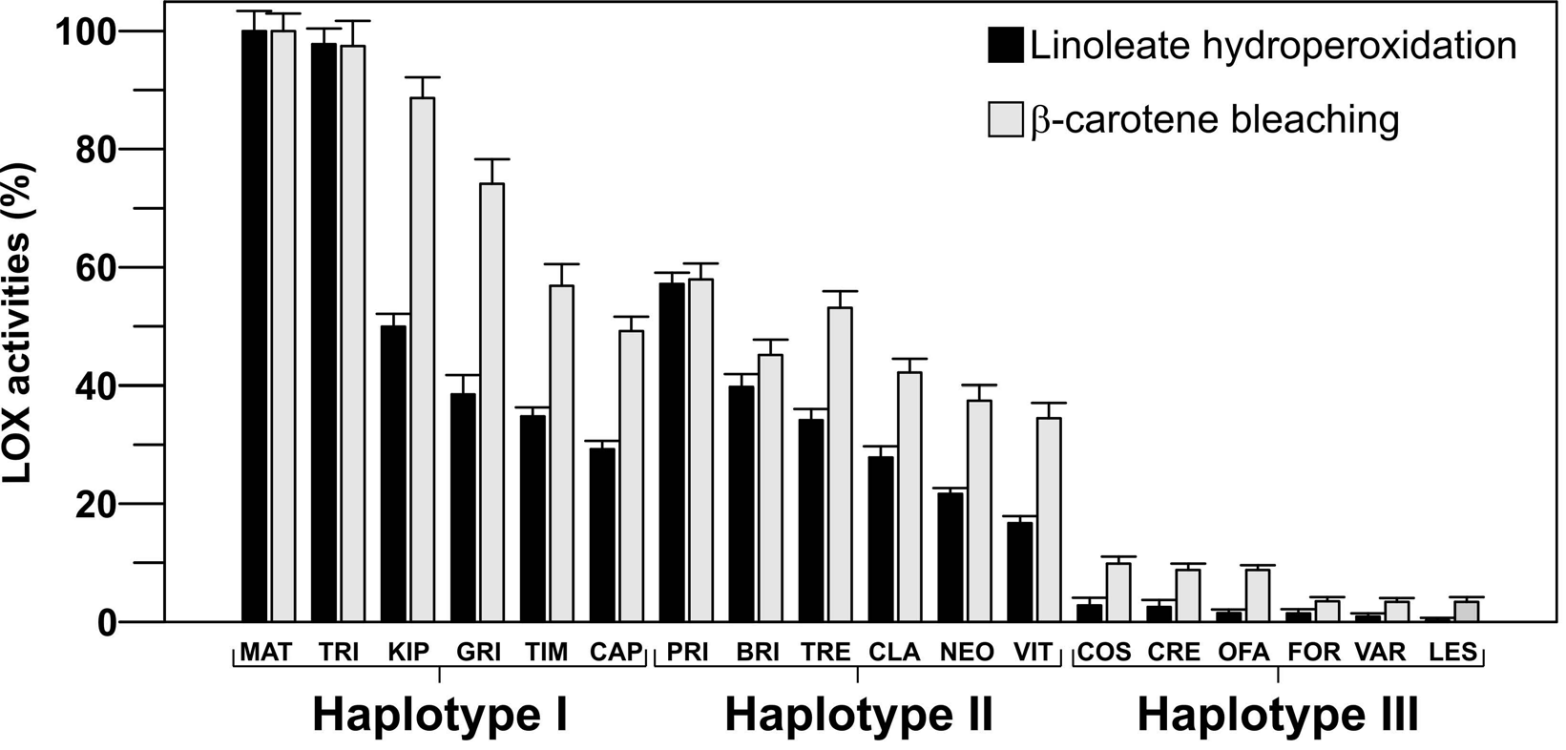
**LOX-catalysed linoleate hydroperoxidation and β-carotene bleaching activities**. Measurements were carried out on crude extracts from the mature grains of the 18 durum wheat cultivars of the germplasm collection as reported in Methods. The specific activity was expressed as a percentage of the highest values obtained for Matarese taken as 100%. For cultivar abbreviations, see legend to Figure 3B. Data are means of the three field replications ± S.D. (n = 3).

We also evaluated LOX activity in crude protein extracts of mature grains from the same 18 durum wheat cultivars used for the gene and transcript analyses. This analysis was based on determination of LOX-catalysed linoleate hydroperoxidation and β-carotene bleaching, the latter being responsible for pigment loss during pasta processing. Both of the enzymatic activities were evaluated on the three field replications of each cultivar and the data obtained were expressed as enzymatic units (E.U.)/g of dry weight (Table 2) and as percentages of the highest values obtained for the cultivar Matarese taken as 100% (Figure 6).

As shown in Figure 6, the two activities followed roughly the same trend among cultivars, as high linoleate hydroperoxidation activity was always associated with high β-carotene bleaching activity. These data are in agreement with strict association between these two LOX-catalysed reactions [5]: the higher the rate of intermediate radicals generation during the LOX-catalysed linoleate hydroperoxidation, the higher the rate of pigment oxidation. As shown in Table 2, the haplotype I cultivars showed the highest LOX activities, with mean values of 4.60 ± 2.38 E.U./g of dry weight for linoleate hydroperoxidation, and 5.76 ± 1.50 × 10^-2 ^E.U./g of dry weight for β-carotene bleaching activity. Intermediate LOX activities were seen in the haplotype II cultivars, which showed linoleate hydroperoxidation activity of 2.59 ± 1.08 E.U./g of dry weight and β-carotene bleaching activity of 3.34 ± 0.65 × 10^-2 ^E.U./g of dry weight. Low LOX activities characterised the haplotype III cultivars, with mean enzymatic activities of 0.12 ± 0.09 E.U./g of dry weight and 0.47 ± 0.23 × 10^-2 ^E.U./g of dry weight for linoleate hydroperoxidation and β-carotene bleaching, respectively. Statistical analysis revealed that the three haplotypes are significantly different (*P < 0.05*) with respect to both linoleate hydroperoxidation and β-carotene bleaching activity.

**Figure 7:**
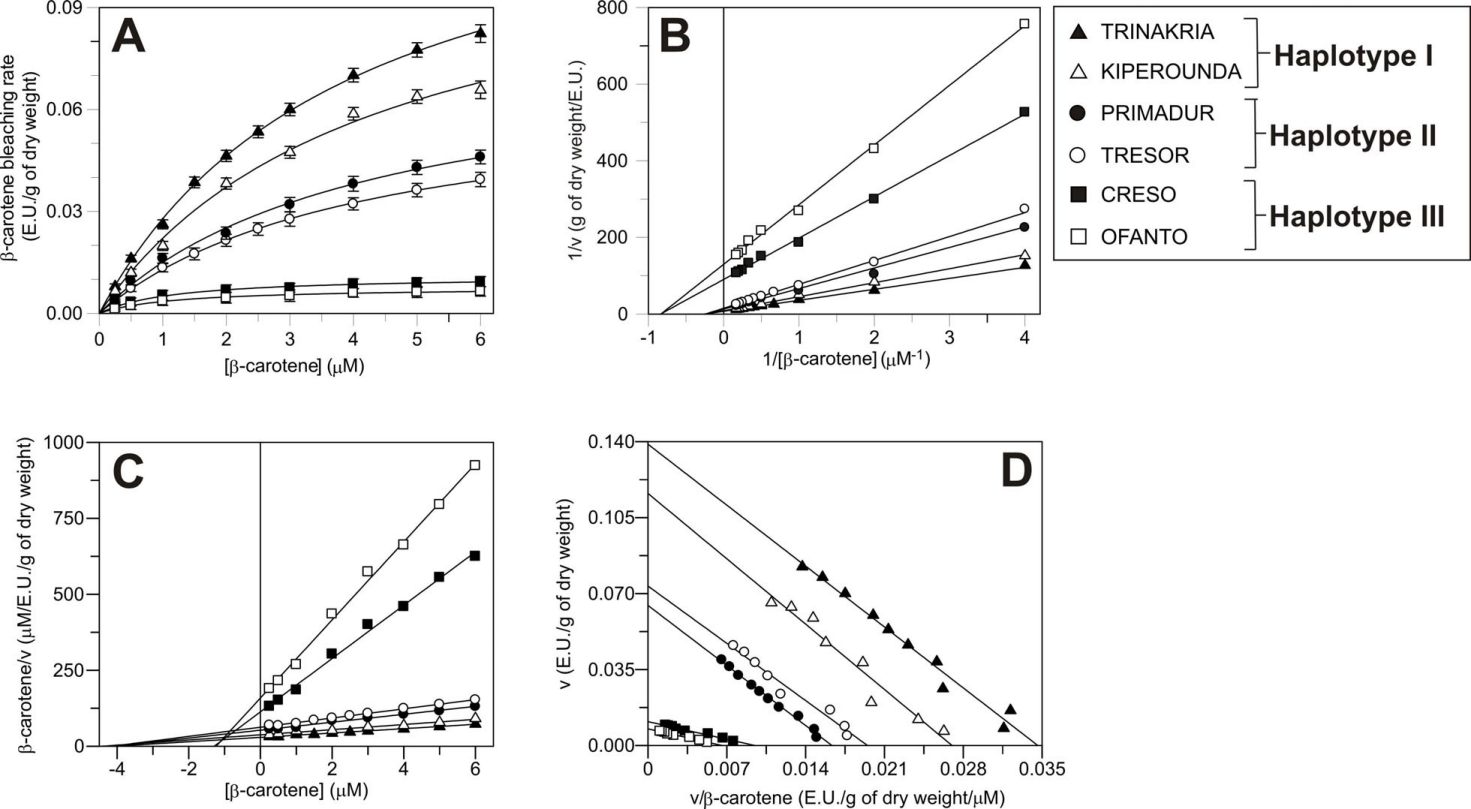
**Dependence of the LOX-catalysed bleaching reaction on β-carotene concentration**. Measurements were carried out as described in Methods, with β-carotene concentrations varied as indicated. Data are presented as Michaelis-Menten (A), Lineweaver-Burk (B), Hanes (C) and Eadie-Hofstee (D) plots. In the Michaelis-Menten plot data are expressed as means of the three field replications ± S.D. (n = 3).

To determine whether in addition to the quantitative differences in the levels of active enzyme, the cultivars that belong to the different haplotypes also had qualitative differences in LOX catalysis, we investigated the kinetic parameters of the LOX-catalysed β-carotene bleaching reaction. This secondary reaction was chosen as it is the one that is directly responsible for the pigment loss during pasta processing. Two cultivars for each haplotype were chosen: Trinakria and Kiperounda for haplotype I, Primadur and Tresor for haplotype II, and Creso and Ofanto for haplotype III. The data obtained are shown in Figure 7. In agreement with previous findings [19], the β-carotene bleaching rate showed a hyperbolic dependence on β-carotene concentration (Figure 7A). Due to the hyperbolic nature of these kinetics, the data were plotted according to Michaelis-Menten [20] (Figure 7A), Lineweaver-Burk [21] (Figure 7B), Hanes [22] (Figure 7C), and Eadie-Hofstee [23] (Figure 7D) plots, and the Km values for β-carotene were determined from each of these plots. As shown in Table 5, the four different plots gave the same Km values. Comparisons among cultivars showed that the haplotype I and haplotype II cultivars shared the same affinity for β-carotene (Km around 4 μM), whereas a significantly different (*P < 0.05*) Km was extrapolated for the cultivars belonging to haplotype III (around 1.2 μM).

**Table 5:** Km values for β-carotene extrapolated from the hyperbolic kinetics and the corresponding linearisations shown in Figure 7

Plot	Km (μM)					
	
	Haplotype I		Haplotype II		Haplotype III	
	Trinakria	Kiperounda	Primadur	Tresor	Cosmodur	Ofanto
Michaelis-Menten	3.99 ± 0.16	4.24 ± 0.55	4.11 ± 0.12	4.18 ± 0.45	1.19 ± 0.11	1.19 ± 0.12
Lineweaver-Burk	4.18 ± 0.25	4.11 ± 0.47	3.99 ± 0.21	4.07 ± 0.19	1.21 ± 0.05	1.24 ± 0.08
Hanes	4.00 ± 0.17	4.26 ± 0.43	4.10 ± 0.15	3.95 ± 0.31	1.26 ± 0.13	1.22 ± 0.10
Eadie-Hofstee	4.01 ± 0.21	4.30 ± 0.40	3.95 ± 0.13	3.96 ± 0.28	1.15 ± 0.07	1.16 ± 0.07
Mean value^a^	4.04 ± 0.19^A^	4.22 ± 0.40^A^	4.03 ± 0.15^A^	4.04 ± 0.29^A^	1.20 ± 0.09^B^	1.20 ± 0.08^B^

^a^Values are expressed as means of the three field replications ± S.D.; the different uppercase letters correspond to significantly different values (*P *< 0.05).

## Discussion

In durum wheat, there are two *Lpx-1 *genes on chromosome 4B: *Lpx-B1.1 *and *Lpx-B1.2*. Evidence has been provided in the literature that deletion of *Lpx-B1.1 *is associated with a strong reduction in LOX activity in semolina [4,10]. This finding is also supported by results obtained in common wheat (*Triticum aestivum *Desf.), which assigned the *Lpx-1 *genes to the group 4 chromosomes [24,25] and, very recently, highlighted the existence of a major QTL for LOX activity on chromosome 4B [26,27], thus confirming a significant effect of this locus on the enzyme activity in wheat seeds.

Despite the relevance of the *Lpx-B1 *locus in pigment degradation during pasta processing, its role in this phenomenon has not yet been fully defined. In the present study, we report the characterisation of the small *Lpx-B1 *gene family identified in a large durum wheat germplasm collection, and relate the distribution and expression levels of the *Lpx-B1 *genes and alleles to the variations in LOX activity in mature grains.

### Characterisation of the small *Lpx-B1 *gene family in durum wheat germplasm

On the basis of the segregation data, we propose that the small *Lpx-B1 *gene family consists of three different *Lpx-B1 *genes. All of these genes are located on chromosome 4BS, in agreement with previous reports [10,11]. In addition to the already known *Lpx-B1.1 *and *Lpx-B1.2 *genes, a new gene designated as *Lpx-B1.3 *was identified, along with three different alleles at the *Lpx-B1.1 *locus. Mapping data from the Creso × Pedroso and Ofanto × Cappelli RIL populations suggested that *Lpx-B1.3 *is a separate locus with respect to *Lpx-B1.2*, even if the two loci are closely linked (2 cM in Creso × Pedroso, and 0.6 cM in Ofanto × Cappelli). Only one recombinant line was found in the Ofanto × Cappelli population, which was between *Lpx-B1.2 *and *Lpx-B1.3*, and was characterised by the absence of both the *Lpx-B1.2 *and *Lpx-B1.3 *bands. Three recombinant lines were found in the Creso × Pedroso population, and one of these was characterised by the absence of both the *Lpx-B1.2 *and *Lpx-B1.3 *bands. These data indicate that *Lpx-B1.2 *and *Lpx-B1.3 *represent separate loci.

Comparisons with the orthologous rice *9-LOX1 *gene allowed determination of the exon/intron structure of the *Lpx-B1 *genes and alleles. Although these were highly conserved across these two species, there was the notable exception of the loss of introns 3 to 5 in the durum wheat genes. In contrast, the maize and sorghum orthologues [GenBank: AF465643 and CM000760, respectively] have maintained introns 3 to 5, but lack introns 6 and 8. All of these cases are precise intron losses that are known to have occurred in the course of the evolution of these plant genomes [28], probably as a consequence of homologous recombination between cDNA and its genomic intron-containing locus [29]. Comparisons among the different LOX-1 genes in grasses strongly suggested that the independent intron loss events probably occurred after the divergence of the different subfamilies from their common ancestor.

As expected, sequence comparisons among the five durum wheat *Lpx-B1 *genes/alleles revealed a high degree of conservation across the alleles at the *Lpx-B1.1 *locus (more than 99% at the nucleotide level; Table 4). In contrast, the sequence identity between *Lpx-B1.2 *and *Lpx-B1.3 *was lower (97.6%; Table 4) and the gene structures highlighted some interesting differences in terms of intron lengths (Figure 2). In particular, while the length of introns was perfectly conserved in the first part of the gene (introns 1 and 2), introns 3 to 6 were all shorter in the HM126469 sequence than in HM126467. Several SNPs and InDel polymorphisms were also detected through comparisons of the corresponding introns in the different *Lpx-B1 *genes and alleles. Here, the length polymorphism was of particular interest, as has been reported previously [10,11], due to the presence in the last intron of the *Stowaway*-type MITE. In agreement with previous findings, a complete MITE was followed by a partial one (including a direct repeat of the second half of the element) in the Lpx-*B1.1a *allele, and there was a partially deleted element in the *Lpx-B1.1b *allele, while in the *Lpx-B1.2 *and *Lpx-B1.3 *genes the MITE was completely absent. These differences are probably the consequences of different insertions and subsequent excision events, which are known to affect the *Stowaway*-type MITE in *Triticeae*, and which often leave partial sequences that represent recognisable footprints of this element (as in the case of the *Lpx-B1.1a *and *Lpx-B1.1b *alleles) [30]. In addition to these small-scale rearrangements, transposable elements are also well known for their ability to cause large-scale rearrangements, including the deletion of sequences adjacent to the MITE [31]. In the light of this, it is feasible that the instability due to the presence of the MITE might be responsible for the large deletion in the *Lpx-B1.1c *allele, and consequently, for its loss of function.

As far as the distribution of the *Lpx-B1 *genes and alleles in the large germplasm collection of durum wheat genotypes is concerned, three distinct pools were identified that corresponded to three different haplotypes (I, II, III). Of note, genotypes released before 1970 included all of these three haplotypes, while all of the genotypes released successively and characterised by having short straw belong to haplotypes II and III. Thus the *Lpx-B1.1b *allele and the *Lpx-B1.3 *gene of the haplotype I are absent in the modern germplasm. The *Lpx-B1 *locus has been mapped to the same chromosomal region as the *Rht-B1b *(Rht1) dwarfing gene [32], which was introduced into durum wheat from the hexaploid wheat cultivar Norin 10 through introgression. Therefore, it is feasible that the distributions of the *Lpx-B1 *genes and alleles have been profoundly affected by the footprint of the selection at the *Rht-B1b *locus. Previous findings by Hessler et al. agree with this hypothesis, as they detected a fragment (J4.2) corresponding to the *Lpx-B1.1b *allele in an old and tall landrance that was grown in north Africa (Jennah Khetifa), and a fragment (C7.1.2) corresponding to the *Lpx-B1.1a *allele in a recent commercial cultivar, Cham 1 [11].

### The role of the *Lpx-B1 *genes and alleles in the determination of LOX-catalysed carotenoid bleaching in mature durum wheat grains

Comparisons between the data obtained at the molecular and biochemical levels show that in mature durum wheat grains, there is a closely coupled relationship between the *Lpx-B1 *transcripts and LOX catalysis. In particular, a strict relationship was detected for each haplotype: (i) at a quantitative level, between the levels of the *Lpx-B1 *transcripts and the levels of LOX activity, which is linearly dependent on the amount of catalytically active enzyme in this tissue; (ii) at a qualitative level, between the types of *Lpx-B1 *transcript and the kinetic behaviour of the LOX-catalysed bleaching reaction.

For this first point, the haplotype I cultivars are characterised by the higher overall levels of transcript, with both *Lpx-B1.1 *and *Lpx-B1.3 *genes expressed at high levels. As these both encode putatively functional LOX-1 isoforms, high values of total LOX activity are also detected in these haplotype I cultivars. Intermediate transcript levels characterise the cultivars of both haplotypes II and III, in which only the allele at the *Lpx-B1.1 *locus is expressed at high levels. However, as the *Lpx-B1.1a *allele gives a putatively functional LOX-1 isoform while *Lpx-B1.1c *does not, haplotypes II and III have intermediate and low LOX activity levels, respectively, with those of haplotype III being ascribable exclusively to the low expression levels of the *Lpx-B1.2 *gene.

There was also a variability in LOX activity among cultivars belonging to the same haplotype, which cannot be explained by a corresponding variability in transcript levels of the *Lpx-B1 *genes and alleles. As there are two other LOX isoforms, LOX-2 and LOX-3, in cereal grains, their contributions to the total LOX activity cannot be excluded. Indeed, Carrera et al. demonstrated that in their population, the *Lpx-B1 *locus accounted for 54% of the variation in LOX activity in semolina [10]; moreover, we have recently demonstrated that the *Lpx-3 *transcript can also contribute to the total activity in mature grains, particularly in high LOX activity cultivars [4]. The involvement of modifications at post-translational levels that may affect differently the functionality of the LOX-1 isoforms encoded by the different *Lpx-B1 *genes and allele cannot also be excluded.

For the second point above, relating to the qualitative level, the high identity shared at the amino-acid level between the *Lpx-B1.1a *and *Lpx-B1.1b *alleles (99.3%) and between the *Lpx-B1.2 *and *Lpx-B1.3 *genes (99.0%) strongly suggests that the two putative LOX-1 isoforms in the haplotype I cultivars share common structural features with those of the haplotype II cultivars. This hypothesis is supported by findings obtained at the biochemical level, as the haplotype I and II cultivars share the same kinetic behaviour, as demonstrated by the comparable Km values for β-carotene that were extrapolated from the corresponding plots. It is important to note that these Km values obtained are in agreement with the Km previously reported for the haplotype II cultivar Tresor (3.3 ± 0.53 μM; [19]), with the small difference probably ascribable to the different enzyme sources used: in the present study, whole-meal extracts were used, instead of the semolina extracts used in the previous study. On the other hand, only one of the two putative LOX-1 isoforms in the haplotype III cultivars is catalytically active, and this results in a kinetic behaviour (Km values of about 1 μM) that is significantly different (*P < 0.05*) from that of both haplotype I and II cultivars.

In line with these data, recent findings by De Simone et al. revealed that the haplotype I cultivar Trinakria and the haplotype II cultivar Primadur shared the same affinity for linoleate in the LOX-catalysed linoleate hydroperoxidation reaction in the crude extract from mature grains [4]. Also in this case, they provided a significantly different affinity for the haplotype III cultivars Cosmodur and Creso. Moreover, haplotype I and II cultivars were found to share a similar broad bell-shaped pH profile, which differed significantly from the narrow peak for the haplotype III cultivars; this thus suggested at least two LOX isoforms in the haplotype I and II cultivars, and the major contribution of a single isoform in haplotype III. In this regard, we have partially purified two LOX isoforms that show different affinities for linoleate from semolina of the haplotype II cultivar Tresor [19].

## Conclusions

In summary, the present study has revealed that there is a small *Lpx-B1 *gene family in durum wheat germplasm, and that the distribution of the *Lpx-B1 *genes and alleles among the genotypes defines three different haplotypes, I, II and III, that are associated with high, intermediate and low LOX activity in mature grains, respectively. Although haplotype I was lost during the breeding of the modern short-straw genotypes, there remains high variability in the modern cultivars for this trait. The findings of this study will allow a precise selection of the genotypes bearing the deleted *Lpx-B1.1 *allele, and the fixing of haplotype III in all breeding lines, which will contribute to significant improvements in durum wheat quality. Further studies aimed at the biochemical characterisation of the purified LOX-1 isoforms that correspond to the different *Lpx-B1 *transcripts detected in mature grains will extend further our understanding of the role of the *Lpx-B1 *small gene family in the determination of LOX activity.

## Methods

### Plant material and growing conditions

The experiments were carried out on a germplasm collection of 85 genotypes of durum wheat, including old (released before 1971), intermediate (released between 1971 and 1990) and modern genotypes (released between 1991 and 2005). This collection was chosen because it includes all genotypes cultivated in Italy and, therefore, represents a large portion of the genetic diversity present in this country. Moreover, information already exist on this collection with respect to the carotenoid content and to the contribution of the *Lpx *genes to the carotenoid loss during pasta processing [4].

The 85 genotypes were grown in the open field in the winter season 2004-2005 at the experimental station of the Cereal Research Centre of Foggia (Italy) on a clay-loam soil (Typic Chromoxerert) with an average organic matter of 1.97% and a pH 7.7. They were arranged in a randomized complete block with three replications. Each experimental unit consisted of a 10.2 m^2 ^plot. Fertilizer applications were made at pre-sowing (36 kg ha^-1 ^N and 90 kg ha^-1 ^P_2_O_5 _as ammonium biphosphate) and top dressing (52 kg ha^-1 ^N as urea) at Zadoks growth stage 2.2 and 3.1, respectively [33]. For each genotype, the vegetative material at the third-leaf stage from the three replications was collected and immediately stored at -80°C, until used for genomic DNA analysis. The seeds were harvested at physiological maturity on 13 June 2005 at about 11% moisture; the three replications were stored at a constant temperature of 4°C and used to obtain the whole-meal (Tecator Cyclotec 1093) used for the expression analysis and enzymatic assays.

### Assessment of the *Lpx-B1 *genes and alleles among genotypes

Genomic DNA was extracted from about 50 mg leaves of each genotype using a Maxwell™ 16 Instrument (Promega) and the apposite kit, following manufacturer instructions. The concentrations of DNA extracted were determined using a QuBit fluorimeter (Invitrogen), and the DNA quality was evaluated by visualisation on agarose gels.

Evaluation of the distribution of the *Lpx-B1 *genes and alleles among the genotypes was performed first using the primer pair LOXA-L1/R1 (Table 1), which has previously been reported to preferentially amplify the *Lpx-B1 *locus [10]. The amplification was carried out on genomic DNA (100 ng) following conditions described by Carrera et al. [10].

The data obtained were also validated through amplification of each gene and allele using the specific primer pairs reported in Table 1. To be certain that the missing bands were not a result of point mutations in the primer annealing site, amplifications of each fragment corresponding to the complete *Lpx-B1.1*, *Lpx-B1.2 *and *Lpx-B1.3 *genes were carried out with three different primer pairs. Due to the high identity between the *Lpx-B1.1a *and *Lpx-B1.1b *alleles, the same primer pairs were used to amplify the corresponding fragments; as the amplification products included the last intron region, the MITE length polymorphism allowed size discrimination of the two amplification products. All of the fragments were amplified from genomic DNA (100 ng) using Go-Taq DNA polymerase (Promega) under the following conditions: preheating at 94°C for 5 min, then 35 cycles of denaturation at 94°C for 1 min, annealing/extension at 72°C for 1 min, followed by final extension at 72°C for 7 min.

The deleted *Lpx-B1.1c *allele was amplified using the same primer pair used for isolation of the full-length gene sequences (Table 1), but under different amplification conditions: preheating at 94°C for 5 min, then 35 cycles of denaturation at 94°C for 1 min, annealing at 62°C for 30 s, extension at 72°C for 1 min, followed by a final extension at 72°C for 7 min; Go-Taq DNA polymerase (Promega) was used. These conditions allowed amplification of only the deleted gene.

All of the PCR products were visualised on agarose gels, cloned into pGEM-T Easy Vector (Promega), and sequenced on both strands using ABI Prism™ BigDye Terminator Cycle Sequencing kits on an ABI PRISM™ 3130xl Genetic Analyser (PE Applied Biosystem).

### Isolation and sequence analysis of the full-length *Lpx-B1 *genes and alleles

**Figure 8:**
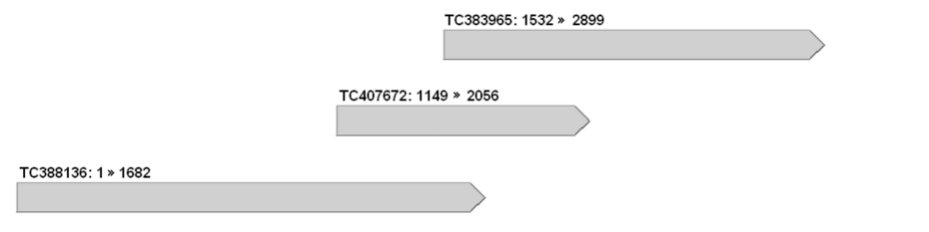
***In silico *contig corresponding to the putative full-length *Lpx-B1 *expressed sequence**. ESTs identified through a BLAST search in the TIGR wheat EST database using the probes of the full-length cDNA sequence of barley LOX-1 [GenBank: L35931] and the partial coding sequences deduced from both DQ474240 and DQ474241.

Primer pairs suitable for amplification of the full-length *Lpx-B1 *genes and alleles were designed on the *in silico *contig reported in Figure 8. This contig was constructed *in silico *by overlapping ESTs found with a BLAST search in the TIGR wheat EST database, using as queries the full-length cDNA sequence of barley *LoxA *[GenBank: L35931] and the partial coding sequences deduced from both DQ474240 and DQ474241. The resulting contig shared 92.5% identity with barley L35931 (from start to stop codon), and in the overlapped regions, 94.9% and 93.1% identity with the predicted partial coding sequences deduced from DQ474240 and DQ474241, respectively. Different primer pairs were designed and tested on genomic DNA, and the one that gave a single PCR product of the expected size was chosen for isolation of the full-length *Lpx-B1 *genes and alleles (Table 1).

The full-length *Lpx-B1 *gene sequences were isolated from the genomic DNA of three cultivars, each of which represented one of the three haplotypes: *Lpx-B1.1b *and *Lpx-B1.3 *from Trinakria (haplotype I), *Lpx-B1.1a *and *LpxB1.2 *from Primadur (haplotype II), and *Lpx-B1.1c *from Ofanto (haplotype III). The amplification reactions were performed using the high fidelity "Long Range" Taq DNA polymerase (Qiagen) and following the manufacturer instructions: preheating at 93°C for 3 min, then 35 cycles of denaturation at 93°C for 15 s, annealing at 62°C for 30 s and extension at 68°C for 7 min. All of the PCR products were visualised on agarose gels, cloned and sequenced on both strands.

The gene sequences have been deposited at GenBank, with the accession numbers of HM126466 (*Lpx-B1.1a*), HM126467 (*Lpx-B1.2*), HM126468 (*Lpx-B1.1b*), HM126469 (*Lpx-B1.3*), and HM126470 (*Lpx-B1.1c*).

### Chromosomal mapping of the *Lpx-B1 *genes and alleles

The positions of *Lpx-B1 *genes and alleles in the durum wheat genome were determined following their segregation in two RIL populations: one that consisted of 123 lines derived from a cross between the durum wheat cultivars Creso and Pedroso, already described by Marone et al. [34], and one that consisted of 121 lines developed from a cross between the durum wheat cultivars Ofanto and Cappelli. The parental lines Creso, Ofanto and Cappelli were all Italian cultivars belonging to the germplasm collection of the Cereal Research Centre, Foggia (Italy), while the parental line Pedroso was a Spanish cultivar provided by the germplasm collection of the CIFA Alameda del Obispo, IFAPA-CICE, Cordoba (Spain). In both cases the lines were obtained by advancing random individual F2 plants to the F7 generation by single-seed descent. These populations were chosen because there were different *Lpx-B1 *genes/alleles in the parental genotypes: HM126467 and HM126470 in Creso and Ofanto, and HM126468/HM126466 and HM126469 in Pedroso and Cappelli. The *Lpx-B1 *genes and alleles were amplified on genomic DNA (100 ng) from the parental genotypes and the RIL population lines using the primer pairs specific for each gene and allele (Table 1), and the same amplification conditions described above.

The genetic maps were constructed using the Kosambi mapping function within the JoinMap 4 software [35], considering a minimum LOD score (log_10 _of the odds ratio) of 4.0. Six hundred and seventeen markers (180 PCR-based, and 437 DArT) were used to construct the Ofanto × Cappelli map, while 500 markers (173 PCR-based and 327 DArT) were used to construct the Creso × Pedroso map [34]. For the DArT assay, DNA from the two mapping populations was subjected to PstI/TaqI digestion and size purification and probed against the durum DArT array at the Pty Ltd (Canberra, Australia; http://www.triticarte.com.au). The goodness of fit for all of the loci was tested to an expected 1:1 segregation ratio, using chi-square analysis. Linkage groups were assigned to chromosomes by comparing the marker positions to previously published durum wheat maps [36-42] and to the hexaploid wheat SSR consensus map [43]. The details of the Ofanto × Cappelli molecular marker map will be published separately; in the present study, only the 4BS linkage group carrying the *Lpx-B1 *loci is presented.

### Isolation and sequence analysis of the full-length *Lpx-B1 *transcripts

The full-length transcripts were isolated from mature grains of the same cultivars used for isolation of the full-length *Lpx-B1 *gene sequences: Primadur, Trinakria and Ofanto. Total RNA was isolated from mature grains using Trizol reagent (Invitrogen), following the manufacturer instructions. The seeds were ground under liquid nitrogen, and to avoid starch contamination, the powder obtained was treated with 50 mM Tris-HCl buffer, pH 9.0, 200 mM NaCl, 1% sarcosil, 20 mM EDTA, and 5 mM DTT, and subjected to phenol-chloroform extraction. The purified samples were then used for Trizol extraction. A DNase treatment step was performed at the end of the extraction to ensure the removal of genomic DNA from the total RNA extracted.

The single-stranded cDNA was synthesised using 200 E.U. of SuperScript™II RNase H^- ^reverse transcriptase (Invitrogen) and a poly(T) primer on 1 μg total RNA, following the manufacturer instructions. The first cDNA strand was used as the template for amplification of the full-length *Lpx-B1 *expressed sequences using the same primer pair used for isolation of the full-length genomic sequences (Table 1) and high-fidelity "Phusion" Taq DNA polymerase (Finnzyme). The PCR conditions were as follows: preheating at 94°C for 5 min, then 35 cycles of denaturation at 94°C for 1 min, annealing at 56°C for 30 s and extension at 72°C for 3 min, followed by final extension at 72°C for 10 min. All the PCR products were visualised on agarose gels, cloned and sequenced on both strands to confirm their identity.

The transcript sequences have been deposited at GenBank, with the accession numbers of HM126471 (*Lpx-B1.1a*), HM126472 (*Lpx-B1.2*), HM126473 (*Lpx-B1.1b*), HM126474 (*Lpx-B1.3*), HM126475 (*Lpx-B1.1c*).

The deduced protein sequences were subjected to bioinformatic analysis. Alignments were carried out using the Vector NTI Suite software (version 9.0; Invitrogen). The physico-chemical parameters of the amino-acid sequences were estimated using the ProtParam tool, which is available at the ExPASy molecular biology server http://us.expasy.org/tools/protparam.html. The conserved domains were determined by using the NCBI Conserved Domain Database http://www.ncbi.nlm.nih.gov/Structure/cdd/cdd.shtml.

### Semi-quantitative RT-PCR analysis

The first cDNA strand was synthesised from total RNA of mature durum wheat grains from 18 cultivars selected from the germplasm collection, and was used as the template for the amplification of fragments corresponding to the *Lpx-B1 *expressed sequences. Normalisation of the RT-PCR reactions was performed by amplifying the wheat TC283024, which was 96% identical to the rice gene for the 17S ribosomal RNA. In this case, single-strand cDNA was synthesised using 200 E.U. of SuperScript™ II RNase H^- ^reverse transcriptase (Invitrogen) and random primers, on 1 μg total RNA.

Amplifications of the fragments corresponding to the *Lpx-B1 *transcript variants were performed using the same specific primer pairs (Table 1) and the amplification conditions used for amplification of the corresponding gene fragments, with the notable exception that 30 cycles were used to ensure semi-quantitative evaluation of the abundance of the mRNA sequences analysed. Amplification of the 17S rRNA was performed using the specific primer pair reported in Table 1, under the following conditions: preheating at 94°C for 5 min, then 30 cycles of denaturation at 94°C for 1 min, annealing at 60°C for 30 s and extension at 72°C for 1 min, followed by final extension at 72°C for 7 min.

### Enzymatic assays

Enzymatic assays of both linoleate hydroperoxidation and β-carotene bleaching were carried out on crude whole-meal extracts. The assays of linoleate hydroperoxidation and β-carotene bleaching activity reported in Figure 6 and in Table 2 were carried out on the same 18 cultivars used for the gene and transcript analyses, while the assays for the kinetic analysis reported in Figure 7 were carried out on two cultivars for each haplotype: Trinakria and Kiperounda for haplotype I, Primadur and Tresor for haplotype II, and Creso and Ofanto for haplotype III. All the assays reported in Figure 6 and 7 and in Table 2 were carried out on the three field replications of each genotype.

Five g of whole-meal were suspended in 10 mL 100 mM sodium phosphate buffer (pH 7.0). The suspension was placed in an ice-water bath for 1 h, and stirred for 1 min at 15 min intervals, followed by centrifugation (twice) at 35,000 × *g *at 4°C for 15 min. The supernatant was stored in an ice-water bath and used on a daily basis. Protein content was determined according to the Lowry method [44], using bovine serum albumin as the standard.

Linoleate and β-carotene solutions were prepared essentially as reported in [19]. Linoleate hydroperoxidation was assayed following absorbance increase at 234 nm, due to the conversion of linoleate into the corresponding hydroperoxide (ε = 28 mM^-1 ^cm^-1^). The reaction mixture consisted of 2 mL 50 mM phosphate buffer (pH 6.6) containing 500 μM linoleate. The reaction was started by the addition of 0.1 mg crude protein extract. One E.U. of linoleate hydroperoxidation activity corresponded to the formation of 1 μmol conjugated diene per min at 25°C. β-carotene bleaching activity was determined by monitoring the absorbance decrease at 460 nm due to the disappearance of β-carotene (ε = 123.5 mM^-1^⋅ cm^-1^) [19]. The reaction mixture consisted of 2 mL 50 mM sodium phosphate buffer (pH 6.6) containing 1 mM linoleate, 6 μM β-carotene, 2.0 μL/mL Tween 20 and 0.33 μL/mL Tween 80. In Figure 7, the β-carotene concentration was varied as indicated. The reaction was started by the addition of 0.5 mg protein extract. One E.U. of β-carotene bleaching activity corresponded to the disappearance of 1 μmol β-carotene per min. All of the assays were carried out at pH 6.6 to simulate conditions that occur during pasta processing. To be sure that these reactions were specifically catalysed by LOX, both linoleate hydroperoxidation and β-carotene bleaching were completely inhibited by addition of propylgallate (500 μM), one of the most specific inhibitors of LOX activity [45] (data not shown).

All of the measurements were carried out using a Perkin Elmer Lambda 650 UV/VIS spectrophotometer. GRAFIT 4.0 software (ERITHACUS, from SIGMA Chemical Co.) was used to analyse the data.

Data from the enzymatic assays were reported as mean of the three field replications ± S.D. Results were subjected to Tukey's HSD (honestly significant difference) test using JMP Starter 8.0 software (SAS. Institute Inc., Heidelberg, Germany) to determine significant differences among haplotypes. An alpha level of 0.05 was used for statistical significance.

## List of abbreviations

E.U.: enzymatic unit; Km: Michaelis-Menten constant; LOX: lipoxygenase; MITE: miniature inverted-repeat transposable element; RIL: recombinant inbred line; S.D.: standard deviation; SNPs: single nucleotide polymorphisms.

## Authors' contributions

AV isolated and characterised the *Lpx-B1 *genes/alleles and transcripts, and performed the mapping and biochemical experiments. VDS carried out the primer design and the expression analysis. AMM developed the design of the mapping experiments and analysed the mapping data. LC provided general guidance for the study. DT conceived, designed, coordinated the study, and drafted the manuscript. AMM, LC and RP contributed to the data interpretation and the writing of the manuscript. All of the authors have read and approved this version of the manuscript.
